# High‐resolution metabolomic profiling of Alzheimer’s disease in plasma

**DOI:** 10.1002/acn3.50956

**Published:** 2019-12-11

**Authors:** Megan M. Niedzwiecki, Douglas I. Walker, Jennifer Christina Howell, Kelly D. Watts, Dean P. Jones, Gary W. Miller, William T. Hu

**Affiliations:** ^1^ Department of Environmental Health Rollins School of Public Health Emory University Atlanta Georgia; ^2^ Department of Environmental Medicine and Public Health Icahn School of Medicine at Mount Sinai New York New York; ^3^ Clinical Biomarkers Laboratory Division of Pulmonary, Allergy Critical Care and Sleep Medicine Emory University Atlanta Georgia; ^4^ Department of Neurology Emory University Atlanta Georgia; ^5^ Center for Neurodegenerative Diseases Emory University Atlanta Georgia; ^6^ Department of Pharmacology Emory University Atlanta Georgia; ^7^ Alzheimer’s Disease Research Center Emory University Atlanta Georgia; ^8^ Present address: Department of Environmental Health Sciences Mailman School of Public Health Columbia University New York New York

## Abstract

**Background:**

Alzheimer’s disease (AD) is a complex neurological disorder with contributions from genetic and environmental factors. High‐resolution metabolomics (HRM) has the potential to identify novel endogenous and environmental factors involved in AD. Previous metabolomics studies have identified circulating metabolites linked to AD, but lack of replication and inconsistent diagnostic algorithms have hindered the generalizability of these findings. Here we applied HRM to identify plasma metabolic and environmental factors associated with AD in two study samples, with cerebrospinal fluid (CSF) biomarkers of AD incorporated to achieve high diagnostic accuracy.

**Methods:**

Liquid chromatography‐mass spectrometry (LC–MS)‐based HRM was used to identify plasma and CSF metabolites associated with AD diagnosis and CSF AD biomarkers in two studies of prevalent AD (Study 1: 43 AD cases, 45 mild cognitive impairment [MCI] cases, 41 controls; Study 2: 50 AD cases, 18 controls). AD‐associated metabolites were identified using a metabolome‐wide association study (MWAS) framework.

**Results:**

An MWAS meta‐analysis identified three non‐medication AD‐associated metabolites in plasma, including elevated levels of glutamine and an unknown halogenated compound and lower levels of piperine, a dietary alkaloid. The non‐medication metabolites were correlated with CSF AD biomarkers, and glutamine and the unknown halogenated compound were also detected in CSF. Furthermore, in Study 1, the unknown compound and piperine were altered in MCI patients in the same direction as AD dementia.

**Conclusions:**

In plasma, AD was reproducibly associated with elevated levels of glutamine and a halogen‐containing compound and reduced levels of piperine. These findings provide further evidence that exposures and behavior may modify AD risks.

## Introduction

Alzheimer’s disease (AD) is a progressive neurological disorder whose onset and progression are influenced by genetic, biological, environmental, and social factors.^1^ There has been considerable progress in characterizing proteomic changes associated with the core pathology of AD (including A*β*42‐rich neuritic plaques and tau‐rich neurofibrillary tangles).^2^, ^3^ However, proteins alone – in the brain or body fluids such as plasma or cerebrospinal fluid (CSF) – are inadequate to characterize metabolic alterations and environmental exposures associated with AD. Targeted metabolomics studies in AD have identified novel risk factors and candidate diagnostic biomarkers,^4^, ^5^ but these approaches are limited in the number of metabolites measured. When coupled with only a modest accuracy in clinical diagnosis^6^ and the presence of pre‐clinical AD in older subjects, these studies are often plagued by false positive or negative findings.

Untargeted metabolomics involves the simultaneous detection of all small molecules in a biofluid – that is, the *metabolome* – without a priori knowledge of the metabolites involved.^7^ One untargeted approach, high‐resolution metabolomics (HRM), enables the measurement of thousands of endogenous and exogenous metabolites over eight orders of magnitude.^8^ We hypothesize that HRM will enable the identification of novel biological and/or man‐made metabolites associated with AD. At the same time, “‐omics” studies in AD research suffer from non‐standardized sample handling, over‐training in a single small cohort, and limited accuracy of the clinical AD diagnosis, with at least 17% of clinically‐probable AD found to have no AD neuropathology on autopsy.^6^, ^9^ Along with inter‐individual variability and technical limitations, these factors contribute to the low replication rates of AD metabolomic profiles.^5^, ^10^

To identify metabolites whose alterations are consistently associated with AD, we designed an HRM study using plasma samples from subjects with normal cognition (NC), mild cognitive impairment (MCI) and AD dementia. We incorporated CSF AD biomarker information associated with the presence or absence of brain AD pathology^11^, ^12^ to achieve high diagnostic accuracy. We then recruited an independent sample of participants to validate our plasma findings, and we used a metabolome‐wide association study (MWAS) approach to identify metabolites consistently altered across the two studies.

## Methods

### Participants

Subjects for both studies were recruited from the Emory Cognitive Neurology Clinic and the Emory Alzheimer’s Disease Research Center. This study was approved by the Emory University Institutional Review Board. All participants or their legal representatives provided written informed consent. Each subject underwent a detailed evaluation including neurological examination and neuropsychological analysis. Subjects with cognitive impairment or dementia also underwent routine blood tests to rule out common reversible causes of cognitive dysfunction, and brain imaging to rule out structural causes of dementia. Subjects were classified as having NC if there was no subjective cognitive complaint and neuropsychological analysis showed normal cognitive functioning according to age, gender, education, and race and as having MCI^13^ or AD dementia^14^ according to NIA‐AA criteria.

### Sample collection

Plasma samples were collected and processed as described previously.^15^ Briefly, 20 mL of whole blood was collected via phlebotomy between 8 am and noon without overnight fasting and centrifuged at 4°C at 1000 x g. Platelet‐rich plasma was immediately removed without disturbing the cellular layers, aliquoted, labeled, frozen, and stored at −80°C until analysis within 2 h of collection. All subjects in the two studies also underwent CSF collection via lumbar puncture with a 24‐gauge atraumatic spinal needle into polypropylene tubes (BD Falcon), immediately aliquoted (0.5 mL), labeled, frozen, and stored at −80°C until analysis within 30 min of collection. CSF AD biomarker analysis was performed as previously described in a Luminex 200 platform, including levels of beta‐amyloid 1‐42 (A*β*42), total tau (t‐Tau), and tau phosphorylated at threonine 181 (p‐Tau_181_).^15^ For inclusion into the study, all NC subjects must have t‐Tau/A*β*42 < 0.39 (not consistent with Alzheimer’s disease), and all AD dementia subjects must have t‐Tau/A*β*42 ≥ 0.39 (consistent with Alzheimer’s disease) based on a previously published CSF autopsy study.^11^ Because only 30–70% of MCI subjects had underlying AD pathology as the cause of their cognitive impairment,^16^ MCI subjects were classified as likely due to AD (MCI‐AD, t‐Tau/A*β*42 ≥ 0.39) or likely due to a suspected non‐AD pathology (MCI‐SNAP, t‐Tau/A*β*42 < 0.39).

### Plasma and CSF HRM analysis

Plasma and CSF samples were prepared for HRM using methods detailed elsewhere.^17^, ^18^, ^19^ Briefly, aliquots were removed from storage at −80°C and thawed on ice, upon which 65 *μ*L of biofluid was added to 130 *μ*L of acetonitrile containing a mixture of stable isotopic standards, vortexed, and allowed to equilibrate for 30 min. Proteins were precipitated by centrifuge (16.1*g* at 4°C for 10 min), and extracts were stored in a refrigerated autosampler. Triplicate 10 *μ*L aliquots were analyzed by reverse‐phase C_18_ liquid chromatography (Dionex Ultimate 3000) and Fourier transform mass spectrometry (Study 1: Thermo Q‐Exactive; Study 2: Thermo Q‐Exactive HF) in positive electrospray ionization mode, resolution (FWHM) of 70,000 (Study 1) or 120,000 (Study 2) and mass‐to‐charge (*m/z*) range of 85–1250.^20^, ^21^ Samples were grouped by matrix, randomized, and analyzed in batches of 20, with a quality control (QC) pooled reference sample included at the beginning and end of each batch. Raw data files were extracted using apLCMS^22^ with modifications by xMSanalyzer,^23^ with each unique mass‐to‐charge (*m/z*) feature defined by *m/z*, retention time, and ion abundance*.* Metabolomics data will be deposited in Metabolomics Workbench (http://www.metabolomicsworkbench.org).

### Statistical analysis

Statistical analyses were performed in RStudio v0.99.486.^24^ Variation in *m/z* feature intensities related to analytical daily batch was removed using ComBat.^25^ Prior to analyses, datasets were filtered to remove features with retention times <30 sec.

Associations of *m/z* features with AD dementia (vs. NC) were assessed using a MWAS approach^26^, ^27^ for features present in >80% of study samples. Since the exclusion and/or imputation of non‐detected intensities can result in biased estimates,^28^ accelerated failure time (AFT) survival models^29^, ^30^ were used to model *m/z* feature intensities as outcomes, which enables the inclusion of all data observations by treating missing values as left‐censored. AFT models were fit using the R package “survival.”^31^, ^32^ For each *m/z* feature, the limit of detection (LOD) was considered to be the lowest detected intensity value. Separately in each study, AFT models, assuming lognormal intensity distributions,^33^ were constructed for each feature wherein diagnostic status (AD vs. NC) was the primary predictor of intensity, adjusted for sex and age; for the *i*th individual,

$$\log\left(\text{Ti}\right)=\mu +\beta_{1}x_{1i}+\beta_{2}x_{2i}+\beta_{3}x_{3i}+\sigma \varepsilon_{i}$$

where log(Ti) is the log‐transformed feature intensity, *X*_1_, *X*_2_, and *X*_3_ represent the predictor variables diagnosis (NC vs. AD dementia), sex, and age (continuous 1‐y change), with coefficients *β*_1_, *β*_2_ *,* and *β*_3_,* μ* is the intercept term, *ε* is the error term, and *σ* is the scale coefficient. The beta coefficient for diagnosis (*β*_1_) can be interpreted as the change in log mean intensity for AD dementia versus NC.

To identify *m/z* features consistently associated with AD dementia, a fixed effects meta‐analysis was conducted using the “meta” R package.^34^ A feature was selected for further characterization if it was associated with AD dementia at false discovery rate (FDR) <0.20 in the meta‐analysis and was associated with AD at *P* < 0.10 in both studies with the same direction of association. An FDR threshold of <0.20 was selected based on previously published metabolomics studies.^35^, ^36^, ^37^ To explore whether these features were iatrogenic metabolites linked to AD medications, regression models were constructed with diagnosis predicting each *m/z* feature, adjusting for sex, age, and detection (yes/no) for three AD medications ([M + H]^+^ for memantine (*m/z* 180.1748) and rivastigmine (*m/z* 251.1753), [M + H]^+^ (^13^C isotope) for donepezil (*m/z* 381.2254)). A feature would be excluded from further analyses if beta coefficients were attenuated following adjustment for AD medications in one or both studies.

Boxplots were created with “NADA,”^38^ an R package for censored data. Correlations of AD‐associated features, CSF markers (e.g., tau, A*β*42) and ApoE genotypes were conducted using Spearman rank‐order correlations. Results were combined in a fixed effects meta‐analysis using the “meta” R package.^34^ Correlations of *m/z* features between plasma and CSF were conducted using Spearman rank‐order correlations in participants with CSF in Study 1.

### Metabolite identification

Detected *m/z* features were matched between studies based upon *m/z* (within 5 ppm) and retention times (within 30 sec) using the “xMSanalyzer” R package.^23^ If one *m/z* feature matched to multiple *m/z* features in the other study, the *m/z* feature with the closest retention time was selected for its match. To cluster *m/z* signals derived from the same metabolites, we used a custom‐designed application that generates a pseudospectrum for a metabolite feature based on intensity correlations and retention time similarities, then predicts adduct, isotope, and/or fragment identities based on the observed mass differences within each cluster.

Database matching by accurate mass was conducted using the Human Metabolome Database (HMDB)^39^ with a tolerance of 5 ppm. Searches were conducted using the predicted adduct/isotope/fragment identity from our algorithm; if the algorithm was unable to predict an identity, the database was searched using common adducts ([M + H]^+^, [M + Na]^+^, [M + K]^+^, [M + H‐H_2_O]^+^, [M + H‐2H_2_O]^+^, [M + ACN+H]^+^, [M + ACN+Na]^+^, [M + 2Na‐H]^+^). Ion dissociation (MS^2^) studies were completed and spectra characterized using CFM‐ID^40^ and Sirius 3.0.^41^ Piperine was confirmed by comparing *m/z* values, retention times, isotopic distribution, and detected adducts to a piperine reference standard (Sigma Aldrich).

## Results

Participant characteristics are presented in Table 1. Study 1 consisted of 41 healthy NC, 45 MCI (20 with MCI‐AD [44% of MCI group]), and 43 AD dementia participants, and Study 2 had 18 NC and 50 AD participants. Plasma metabolomics was run for all participants, while CSF metabolomics was run on a subset of participants in Study 1 (Control, *n* = 25; MCI, *n* = 27; AD dementia, *n* = 26). AD and NC subjects did not differ by sex or age (*P* > 0.20). As expected, AD dementia participants had lower A*β*42 levels and higher t‐Tau and p‐Tau_181_ levels (*P* < 0.0001) and were more likely to have at least one APOE ε4 allele (*P *= 0.003 in Study 1).

**Table 1:** Demographic and clinical data for study samples.

Study 1	Control	AD	MCI	Ctrl vs. AD
	*n* = 41	*n* = 43	*n* = 45	*P* c
Demographics				
Male	11 (27%)^a^	16 (37%)	22 (49%)	0.43
Age (y)	67.5 ± 7.3^b^	65.9 ± 8.8	69.4 ± 6.6	0.36
CSF protein biomarkers				
A*β*42 (pg/mL)	340 ± 137	203 ± 76	218 ± 90	<0.0001
t‐Tau (pg/mL)	44 ± 24	117 ± 70	76 ± 67	<0.0001
p‐Tau_181_ (pg/mL)	32 ± 15	75 ± 32	51 ± 25	<0.0001
t‐Tau/A*β*42	0.14 ± 0.09	0.64 ± 0.39	0.39 ± 0.33	<0.0001
APOE genotypes				
Subjects with data (*n*)	*n* = 18	*n* = 13	*n* = 21	0.003
No ε4 alleles	11 (61%)	1 (8%)	10 (48%)	
One ε4 allele	7 (39%)	8 (62%)	7 (33%)	
Two ε4 alleles	0 (0%)	4 (31%)	4 (19%)	

Study 2	*n* = 18	*n* = 50		*P*
Demographics				
Male	8 (44%)	22 (44%)		1.00
Age (y)	61.2 ± 12.6	65.1 ± 9.3		0.24
CSF protein biomarkers				
A*β*42 (pg/mL)	218 ± 112	143 ± 73		0.01
t‐Tau (pg/mL)	43 ± 30	114 ± 54		<0.0001
p‐Tau_181_ (pg/mL)	27 ± 16	56 ± 27		<0.0001
t‐Tau/A*β*42	0.23 ± 0.13	0.93 ± 0.53		<0.0001
APOE genotypes				
Subjects with data (*n*)	*n* = 16	*n* = 40		0.19
No ε4 alleles	10 (63%)	15 (38%)		
One ε4 allele	5 (31%)	17 (43%)		
Two ε4 alleles	1 (6%)	8 (20%)		

a *N* (%) (all such values).

b Mean ± SD (all such values).

c *P*‐values for control versus AD comparisons from *t*‐tests (continuous variables) or chi‐square tests (categorical variables).

### MWAS of plasma metabolites

HRM profiling of plasma yielded 2320 and 3855 unique *m/z* features in Study 1 and Study 2, respectively. To identify metabolites reproducibly associated with AD dementia, we focused on the subset of 968 *m/z* features that were common to both studies. Within each study, we conducted an MWAS of AD, and we summarized the associations by conducting a meta‐analysis. After adjustment for sex and age, we identified four *m/z* features consistently associated with AD dementia (meta‐analysis FDR < 0.20 and *P* < 0.10 in each study; see extended results with relaxed significance thresholds in Table S1). The feature with the strongest association with AD, *m/z* 251.1753, matched the mass of the [M + H]^+^ adduct of rivastigmine, an AD medication. To verify that the three remaining *m/z* features were associated with AD independently of medications, we constructed models for these features adjusting for diagnosis, sex, age, and detection (yes/no) of three common AD medications identified in the unfiltered datasets (rivastigmine, memantine, and donepezil). Associations between AD and the three *m/z* features were not attenuated after adjustment for medication status, suggesting that their associations were not explained by medication use.

We characterized the identities of the three *m/z* features (Table 2; Table S2). The *m/z* feature 129.0661 was identified by MS^2^ as glutamine. While MS^2^ fragmentation for *m/z* 246.9550 was successful, the MS^2^ spectrum did not have a spectral library match, and *m/z* 349.1515 could not be identified with MS^2^ due to low abundance. The most likely accurate mass database match in HMDB for *m/z* 349.1515 was piperine, which is found in black pepper, *Piper nigrum*.^42^ The identification of *m/z* 349.1515 as piperine was confirmed by accurate mass and retention time matching to a reference standard. The MS^1^ and MS^2^ spectra for *m/z* 246.9550 contained peak patterns consistent with the presence of a halogen isotope (chlorine [Cl] or bromine [Br]). The feature had no matches in HMDB, but a METLIN^43^ search returned nine compound matches, all classified as toxicants. Among these features, glutamine was positively correlated with CSF t‐Tau and p‐Tau_181_ levels and the number of ApoE ε4 risk alleles, and piperine and *m/z* 246.9550 correlated negatively and positively with p‐Tau_181_ levels, respectively (Table 3).

**Table 2:** Non‐medication plasma metabolite features reproducibly associated with AD from MWAS.

Feature			Study 1		Study 2		Meta‐analysis		
*m/z* a	RT^a^	Metabolite	Est (SE)	*P*	Est (SE)	*P*	Est (SE)	*P*	FDR
129.0661	89	Glutamine	0.22 (0.11)	0.04	0.31 (0.13)	0.02	0.25 (0.08)	0.002	0.07
246.9550	127	Unknown	0.41 (0.17)	0.02	0.38 (0.21)	0.07	0.40 (0.14)	0.003	0.08
349.1515	80	Piperine	−0.59 (0.31)	0.06	−0.89 (0.49)	0.07	−0.68 (0.27)	0.01	0.18

a *m/z* and retention time (RT, in seconds) reflect the mean values in Studies 1 and 2.

**Table 3:** Spearman correlations^a^ of AD‐associated non‐medication plasma features with CSF protein biomarkers of AD and APOE‐ε4 genotype.

	A*β*42 (pg/mL)	t‐Tau (pg/mL)	p‐Tau_181_ (pg/mL)	APOE (number of ε4 alleles)
Glutamine	−0.15*	0.21^†^	0.18^†^	0.33^†^
*m/z* 246.9550	−0.16^†^	0.13	0.19^†^	0.09
Piperine	−0.01	−0.16*	−0.17^†^	0.04

a Correlations reflect results from a fixed effects meta‐analysis of the partial Spearman correlations, adjusted for sex and age, between the listed variables in Studies 1 and 2; *P < 0.10; ^†^ *P* < 0.05.

### Metabolite alterations in MCI

**Figure 1:**
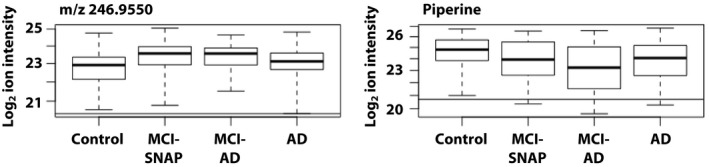
Boxplots of plasma features altered in MCI and AD in Study 1. Figure displays boxplots of log_2_‐transformed feature intensities by diagnosis for metabolites altered in both MCI and AD in Study 1. Horizontal lines show the lowest detectable ion intensity for the corresponding feature.

We explored whether the three metabolites were altered in MCI subjects in Study 1. Two metabolites, *m/z* 246.9550 and piperine, were associated with MCI (vs. NC) in AFT models adjusted for sex and age, both in the same direction as their associations with AD dementia. Stratification by MCI‐SNAP and MCI‐AD did not reveal differences in the feature intensities between the two subgroups (Fig. 1).

### Correlation of metabolites between plasma and CSF

To determine the plausibility of plasma metabolites influencing the brain, we checked whether the features were detectable in CSF. We identified matches for glutamine and *m/z* 246.9550; plasma and CSF levels were modestly correlated for glutamine (*rho* = 0.26, *P* = 0.03) but not for *m/z* 246.9550 (*rho* = −0.04, *P* = 0.72). CSF levels of these two features were not associated with AD (*P* > 0.05; data not shown).

## Discussion

Untargeted metabolomics has the promise to identify novel compounds involved in AD pathogenesis, but inconsistencies in clinical diagnoses, sample handling protocols, and analytical methods affect the generalizability of metabolomics studies. Here, we identified plasma and CSF metabolites associated with biomarker‐supported AD in two studies that we recruited and analyzed independently. In plasma, we replicated associations with glutamine, a previously‐identified metabolite, and we found novel metabolites increased (unknown halogen‐containing compound) and decreased (piperine) in AD dementia. Glutamine and the halogen‐containing compound were also detectable in CSF, supporting their entry into or synthesis in the central nervous system. In sum, our findings suggest that AD dementia is associated with reproducible alterations of plasma metabolites of endogenous and exogenous origin.

Previous studies have examined untargeted metabolomic profiles of AD dementia with varying approaches and results (summarized in Table 4). Several untargeted metabolomics and lipidomics studies have identified AD‐associated phospholipid alterations.^44^, ^45^, ^46^, ^47^ In most of these studies, the primary outcome was classification accuracy, which relies on machine learning algorithms that run the risk of overfitting. Only one study^48^ included Discovery and Validation studies similar to our design, which identified three metabolites across the two samples. To the best of our knowledge, our study is the first to employ HRM in two independent studies to identify metabolites reproducibly associated with biomarker‐supported AD dementia. However, replication in another geographical region is needed for generalization of our findings beyond the southeastern United States.

**Table 4:** Summary of untargeted metabolomics studies of AD in human biofluids.

Study	Diagnostic groups	Replication cohort	Confirmation of AD pathology	Specimen(s)	Platform	Analytical approach	Metabolite results
Orešič et al. 2011^47^	MCI (*n* = 143), AD (*n* = 47), Control (*n* = 46)	None	Imaging, CSF biomarkers	Serum	GC‐MS	Penalized GLM, logistic regression	Molecular signature of AD of three metabolites (PC (16:0/16:0); unidentified carboxylic acid; 2,4‐dihydroxybutanoic acid)
Ibáñez et al. 2012^10^	AD (*n* = 25), MCI‐AD (*n* = 13), MCI‐SNAP (*n* = 24), Control (*n* = 23)	None	Imaging, CSF biomarkers	CSF	CE‐MS	PCA, LDA	14 key metabolites that predicted progression to AD (including choline, dimethylarginine, arginine, valine, proline, serine, histidine, creatine, carnitine, and suberylglycine)
Trushina et al. 2013^72^	MCI (*n* = 15), AD (*n* = 15), Control (*n* = 15)	None	None	Plasma, CSF	LC–MS	ANOVA, PCA, OPLS‐DA	342 plasma and 351 CSF features altered across AD, MCI, and control groups (22% putatively identified)
Motsinger‐Reif et al. 2013^54^	AD (*n* = 40), Control (*n* = 38)	None	CSF biomarkers	CSF	GC–MS, LC–MS	Stepwise logistic regression	Two discriminant features for AD vs. control
Cui et al. 2014^48^	AD (*n* = 46), Control (*n* = 37)	AD (*n* = 63), Control (*n* = 67)	None	Serum, urine	LC–MS	OPLS‐DA, ANCOVA, logistic regression	Three metabolites (serum palmitic amide and lysoPC(18:2), urine 5‐L‐glutamylglycine) with consistent prediction across studies
Graham et al. 2015^73^	MCI (*n* = 16), MCI‐AD (*n* = 19), Control (*n* = 37)	None	None	Plasma	LC–MS	OPLS‐DA, t‐tests	263 metabolites altered in MCI vs. control, 162 metabolites altered in MCI‐AD vs. control (putatively identified)
Morris et al. 2018^74^	AD (*n* = 64), Control (*n* = 62)	None	None	Serum	LC–MS	PLS‐DA, Mann–Whitney *U*‐tests	Poor classification of AD vs. control; ability to distinguish type 2 diabetes patients in controls but not in AD
Pena‐Bautista et al. 2019^75^	MCI‐AD (*n* = 29), Control (*n* = 29)	None	Imaging, CSF biomarkers	Plasma	LC–MS	Elastic net	53 discriminant features; confirmed identity for choline
Habartová et al. 2019^76^	AD (*n* = 20), Control (*n* = 13)	None	Imaging	Plasma	LC–MS	LDA	Seven features altered in AD vs. control (putatively identified)

AD, Alzheimer’s disease; MCI, mild cognitive impairment; SNAP, suspected non‐Alzheimer’s pathophysiology; CSF, cerebrospinal fluid; CE‐MS, capillary electrophoresis‐mass spectrometry; LC–MS, liquid chromatography‐mass spectrometry; GC‐MS, gas chromatography‐mass spectrometry; PCA, principal component analysis; LDA, linear discriminant analysis; PET, positron emission tomography; ANOVA, analysis of variance; GLM, generalized linear model; OPLS‐DA, orthogonal partial least squares discriminant analysis; ANCOVA, analysis of covariance; PLS‐DA, partial least squares discriminant analysis.

Consistent with previous targeted metabolomics studies,^4^, ^49^, ^50^, ^51^, ^52^, ^53^, ^54^, ^55^ we identified elevated plasma glutamine in AD dementia. In our studies, glutamine was positively associated with APOE‐ε4 status and CSF t‐Tau and p‐Tau_181_ levels. Glutamine is a precursor to several excitatory (glutamate and aspartate) and inhibitory (neurotransmitter *γ*‐amino butyric acid, or GABA) neurotransmitters.^56^ Previous studies found that mice with knocked‐in apoE *ε*4 have elevated brain glutamine^57^ and greater susceptibility to excitotoxicity^58^ than APOE *ε*3 knock‐in mice. However, human studies examining glutamine levels in AD dementia compared to NC have generated mixed findings.^48^, ^55^, ^59^ Importantly, glutamine can readily pass through the blood‐brain barrier, and we found a modest correlation between plasma and CSF glutamine levels. While it is possible that memantine (a non‐competitive NMDA receptor antagonist) might influence glutamine levels, effect estimates for plasma glutamine and memantine predicting AD dementia were unchanged in models containing both metabolites simultaneously, suggesting these metabolites were independently related to AD. Thus, our current findings add to the body of evidence implicating glutamine dysregulation and excitotoxicity in AD. Future studies should investigate whether plasma glutamine can serve as a biomarker to identify AD patients susceptible to excitotoxicity.

Piperine, which was found at reduced levels in AD, is a bioactive dietary compound found at high levels in black pepper (*Piper nigrum*).^60^ The compound was negatively associated with CSF p‐Tau_181_ and was also reduced in MCI. Piperine has a range of physiological effects, including antioxidant,^61^ antinflammatory,^62^ and anti‐secretase^63^ activities directly relevant to AD. Piperine has been shown to be neuroprotective in AD mouse models,^64^, ^65^, ^66^, ^67^, ^68^ but to our knowledge, this is the first report of an association between piperine and AD in a human study. Although we are unable to draw conclusions about the direction of the association, these findings warrant further investigation given the alkaloid’s low risks and costs, as well as its ability to enhance absorption of other neuroprotective compounds.^69^, ^70^

Identification of *m/z* features remains a bottleneck in HRM. Identification is particularly difficult for low‐abundance metabolites since MS^2^ spectral matching is challenging^71^ and spectral information may not be present in public databases for less‐common compounds. For example, *m/z* 246.9550 matched nine toxicants in METLIN, but none had actual or in silico MS^2^ spectra, and only a small proportion had standards that were available for purchase. However, the metabolite’s association with AD dementia in two independent study samples and its detectable CSF levels support the need for future work to identify the chemical and examine its role in AD susceptibility or pathogenesis.

Our study had several strengths, including the inclusion of multiple biofluids, two independent samples, and application of the HRM platform. Among the three non‐medication metabolites, two were associated with MCI in Study 1 regardless of cause, suggesting a role in brain vulnerability or degeneration not specific to AD. We also acknowledge limitations beyond those inherent to observational studies, sample size concerns, and generalizability beyond the study’s geographic region. First, it is possible that AD treatment alters non‐neurological metabolic pathways. We could not confirm the identities of two features through MS^2^. We did not have lifestyle history to corroborate past exposures, and we did not have sufficient information (e.g., half‐life) on their associated metabolites to build a temporal relationship between exposure and disease onset. Additionally, the analysis of non‐fasting plasma samples may have introduced noise into the metabolite measurements. Nevertheless, our HRM workflow identified endogenous and environmental metabolites reproducibly altered in AD dementia, providing confirmatory and novel findings for hypothesis generation for future in vitro mechanistic studies and in vivo observational studies.

## Conflict of Interest

The authors declare no actual or potential conflicts of interest.

## Supporting information


**Table S1**. Metabolites associated with AD dementia in Studies 1 and 2 with *P* < 0.20 and consistent direction of association.

**Table S2.** Summary of MS^1^ and MS^2^ results for *m/z* 129.0667, 246.9550, and 349.1515.
