# Supplementary material for: High‐resolution metabolomic profiling of Alzheimer’s disease in plasma
Source: Ann Clin Transl Neurol. 2019 Dec 11;7(1):36–45. doi: 10.1002/acn3.50956 (PMC6952314; doi:10.1002/acn3.50956)
Supplement: Supplementary file 2 — Table S2. Summary of MS1 and MS2 results for m/z 129.0667, 246.9550, and 349.1515. [file ACN3-7-36-s002.docx]

**Summary of MS^1^ and MS^2^ results for *m/z* 129.0667, 246.9550, and 349.1515**

| ***m/z*** | **Time (sec)** | **Identification** | **Annotation confidence level** | **MS^1^ masses** | **MS^2^ characteristic fragments** | **Interpretation** |
| --- | --- | --- | --- | --- | --- | --- |
| 129.0667 | 109.2 | L-Glutamine | Level 2a, probable structure by library search | 129.0660 (M+H-H_2_O); 130.0692 (M+H-H_2_O [^13^C]) | 83.0566; 56.0498; 84.0450 | The *m/z* matched the M+H-H_2_O adduct of glutamine. MS^2^ fragments were consistent with three METLIN and MassBank listed MS^2^ spectra for glutamine. While isoglutamine and D-glutamine would have identitical masses and fragmentation patterns, both would be present (if at all) at levels too low to produce useable spectra. Based on the presence of the MS^2^ masses, the isotopic distribution and fragments consistent with glutamine, this metabolite is verified with Level 2a confidence. Comparison to a reference standard would increase confidence to Level 1. |
| 246.9550 | 125.6 | n/a | n/a | 246.9550 (M+H-H_2_O); 286.9241 (M+Na); 288.2389 ([M+2]+Na) | 142.9673; 144.9382; 162.9491; 164.9461; 215.9783; 224.9032 | The *m/z* matched the M+H-H_2_O adduct of parent ion with *m/z* of 263.9577. MS^2^ fragmentation was successful but did not match any METLIN or MassBank spectra. |
| 349.1515 | 86.7 | Piperine | Level 1, match reference standard by accurate mass and retention time | 349.1525 (M+ACN+Na); 308.1261 (M+Na); 286.1441 (M+H); 350.1559 (M+ACN+Na [^13^C]); 324.0999 (M+K); 287.1470 (M+H [^13^C]) | n/a | The *m/z* matched the M+ACN+Na adduct of piperine. MS^2^ fragmentation was unsuccessful, but a Level 1 verification was obtained by matching to a piperine reference standard by two orthogonal properties (accurate mass and retention time). |

***m/z* 129.0661**


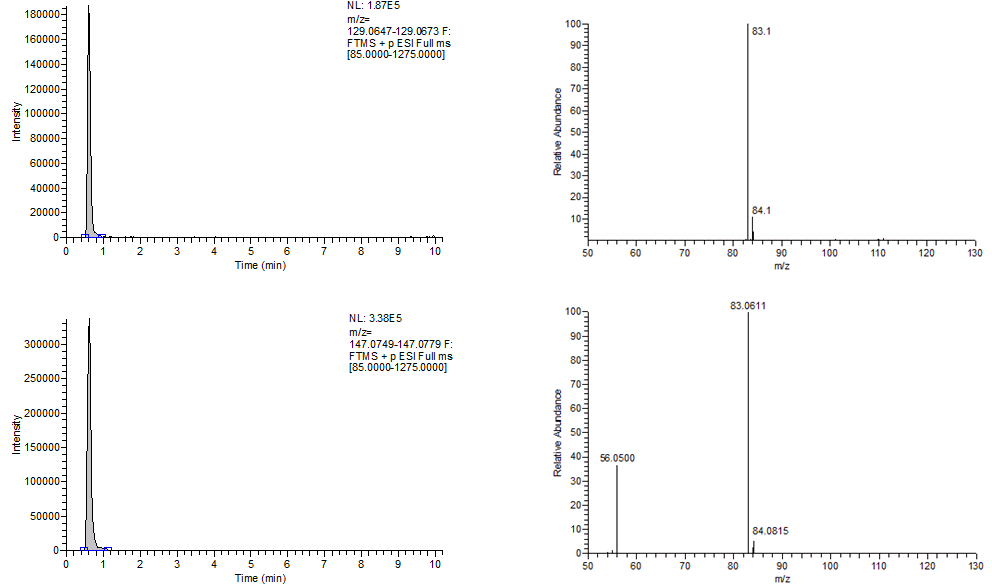


**C**

**A**

**D**

**B**

**Ion dissociation analysis of the [M+H-H_2_O]^+^ glutamine adduct.** A) Extracted ion chromatogram for *m/z* 129.0660, the [M+H-H_2_O]^+^ adduct of glutamine. B) Extracted ion chromatogram for [M+H]^+^ form of glutamine, which showed retention consistent with *m/z* feature 129.0660. C) Collision induced dissociation (CID) of *m/z* 129.0660. D) Higher energy collision dissociation (HCD) spectra for *m/z* 129.0660.

***m/z* 246.9550**

**B**

**A**

1. MS^1^ pseudospectrum for *m/z* 246.9550 in Study 1. B) MS^2^ spectrum for *m/z* 246.9550 in Study 1.

***m/z* 349.1515**

**B**

**A**

MS^1^ pseudospectra for *m/z* 349.1515 in A) Study 1 and B) Study 2.

Comparison of MS^1^ and MS^2^ results for a piperine reference standard and piperine *m/z* features detected in the plasma samples.
